# LifeWatchGreece: Construction and operation of the National Research Infrastructure (ESFRI)

**DOI:** 10.3897/BDJ.4.e10791

**Published:** 2016-11-01

**Authors:** Christos Arvanitidis, Eva Chatzinikolaou, Vasilis Gerovasileiou, Emmanouela Panteri, Nicolas Bailly, Nikos Minadakis, Alex Hardisty, Wouter Los

**Affiliations:** ‡ Institute of Marine Biology, Biotechnology and Aquaculture, Hellenic Centre for Marine Research, Heraklion, Greece; § Institute of Computer Science (ICS), Foundation of Research and Technology - Hellas (FORTH), Heraklion, Greece; | Computer Science & Informatics, Cardiff University, Cardiff, United Kingdom; ¶ Faculty of Science, University of Amsterdam, Amsterdam, Netherlands

## Introduction﻿

Research Infrastructures (RIs) are considered to be tools for science and operate in the form of facilities, resources and services. They promote technological development and provide an accelerator for the advancement of knowledge by offering unique research services to all interested users and stakeholders to enable top-level research in all possible scientific disciplines: from social sciences to astronomy and from genomics to nanotechnologies. High level research often cannot be carried out with only the existing facilities of any institute and frequently depends on large scale RI on national or international scale. Examples of such RIs may include large-scale facilities such as collections, special habitats, libraries, databases, biological archives, clean rooms, mesocosm tanks, integrated arrays of small research installations, high-capacity/high-speed communication networks, high performance computing clusters (HPC, supercomputers), data infrastructure, laboratories, research vessels, satellite and aircraft observation facilities, coastal observatories, telescopes, synchrotrons and accelerators (Commission Staff Working Document 2013). The RIs may be: (a) “single-sited”, which are installed at a single location and based on a single resource, (b) “distributed”, which are applicable to a network of resources or, (c) “virtual”, where services are provided electronically, either as “web services” or “mashups” (e.g., web application hybrids combining content from more than one source into, for example, a virtual laboratory). According to the European Strategy Forum on Research Infrastructures (ESFRI), “*RIs are facilities, resources or services of a unique nature identified by European research communities to conduct top-level research activities in all fields*” (European Commission 2013). ESFRI has produced since 2008 and every two years a RI Roadmap with new infrastructure initiatives that should be implemented to fill existing gaps, promote cutting edge science and make Europe more competitive.

The life cycle of the RIs in the ecosystem of ESFRI is characterized by four phases: (a) Development of a scientific and technical plan, either at national scale or at the European level in the course of the EC Framework Programmes (FPs). Such a plan may subsequently be submitted to the ESFRI roadmap for admission as an ESFRI project; (b) Once admitted with a place on an ESFRI Roadmap, the initiative becomes eligible for a competitive “preparatory phase” with project funding that deals with the refinement of their scientific and technical plan, their scheme of governance, the definition of their legal status and their financial sustainability. This phase ends by establishing a legal RI entity, for example with the submission and subsequent possible approval of an ERIC legal identity (European Research Infrastructure Consortium) by the EC; (c) The next phase includes the “construction and implementation” of the RIs and their operation as legal entities, once their legal personality is established, for example when an ERIC has been signed by the participating Member States (MSs). The RIs are expected to operate at that stage over several decades and to advance through reforms and reorganization whenever needed during their operational phase, or otherwise to enter to the next phase; (d) RIs that cannot function any longer for whatever reason have to be decommissioned or re-orientated (upgraded) or become part of another RI or any other type of organization (European Strategy Forum on Research Infrastructures 2016).

The idea of LifeWatch as a RI has been developed in the 1990s and early 2000s, with the support of EU Networks of Excellence (NoE) and other similar EU funded projects related to biodiversity and ecosystem dynamics and functioning (e.g., MarBEF, ALTER-Net, MGE, EUROCEANS, LTER, CETAF, ENBI, BioCASE, SYNTHESYS and EDIT). These initiatives paved the way for the LifeWatch RI indicating that breakthroughs in biodiversity science will be largely promoted by a sufficiently large European-scale RI on biodiversity data and data observatories. Such an RI should provide advanced capabilities for data integration, analysis and simulations to the scientific community. A few countries submitted this concept to ESFRI, resulting in its selection for the first ESFRI Roadmap 2006. The above concept was successively formally detailed with a construction and financial plan during the preparatory phase of LifeWatch (2008-2011) which was funded as a specific project of the 7^th^ FP. This project prepared a ‘Master Plan’, which describes to a certain detail both the construction elements of LifeWatch RI and associated costs (European Commission 2012). This Master Plan has been further refined by taking into account: (a) a realistic funding scheme by the participating MSs, (b) the available research facilities, currently contributed to the RI by the different countries that have expressed their interest to participate in LifeWatch, and (c) the suggestions and comments provided by both the ESFRI high-level Assessment Expert Group and the ESFRI Strategic Working Group on Environment (European Commission 2015). By the end of the preparatory project a number of countries agreed to cooperate by signing a Letter of Intent, which also commissioned to establish a Stakeholders Board with national representatives and serving as a provisional governing body. The Stakeholders Board reviewed the results of efforts in a so-called start-up phase, temporarily funded by Spain, Italy and Netherlands. The start-up phase prepared all necessary steps for a transition to the ERIC status, including for example Rules for Executive Bodies, Staff Rules, and Accounting Rules. Apart from this governing body, four LifeWatch operational meetings have been held in Lecce (Italy, November 2013), Granada (Spain, February 2014), Crete (Greece, July 2014) and Málaga (Spain, February 2015). In January 2014, the statutes and governance scheme of the Master Plan have been modified, following the detailed texts from the start-up phase and the comments provided by the EC during the step-1 submission of LifeWatch ERIC application back in July 2013. The LifeWatch ERIC application (step-2) has been submitted from the agreed LifeWatch Statutory Seat in Spain by the Spanish Secretary of State for Research, Development and Innovation to the EC in July 2016, and its final version has been sent back to the EC in September 2016. LifeWatch ERIC is now approved by the ERIC Committee and remains to be published in the Official Journal of the EU (Official Gazette) by the end of 2016. As stated in its statutes: “*The main task of LifeWatch ERIC shall be to establish and operate the infrastructure and information systems necessary to mobilise and integrate data and algorithms for biodiversity and ecosystem research, and to provide analytical capabilities*”.

The founding LifeWatch ERIC MSs are Spain, Belgium, Greece, Italy, Slovenia, and Netherlands. Portugal and Romania have confirmed their willingness to become founding members of this infrastructure as well, while some other MSs initially intend to become observers since they already have national LifeWatch activities. Spain, Italy and Netherlands agreed for extra investments in the central LifeWatch Common Facility while all MSs also invest with in-kind contributions in their distributed LifeWatch Centres.

The purpose of this Collection of papers is to describe the construction and operation phase of the Greek LifeWatch distributed centre: LifeWatchGreece.

## Co﻿ncept﻿

The ingredients for the successful construction and implementation of a RI are: (a) the human network, which includes not only its developers but also its stakeholders and the final users of its facilities and services, that should feel a sense of scientific or policy “ownership” of the RI, (b) the physical installations, where the facilities and services are based, (c) the equipment, which for the LifeWatch RI primarily refers to computer hardware (Los et al. 2010), (d) the software reflecting capabilities, e.g. for data integration, analysis and modeling, and (e) the data (Lee and Gurney 2014). LifeWatch ERIC integrates all of the above components in Virtual Research Environments (VREs), which are composed by highly specialized web services such as electronic services (e-Services) and virtual laboratories (vLabs). In LifeWatchGreece RI, two types of such web services are provided: (a) “e-Services, which allow the user to seek answers for the ‘what’, ‘where’ and ‘when’ type of questions”, and (b) “vLabs, which help scientists and other end users to answer the ‘why’ and ‘how’ questions”. Working with vLabs, as demonstrated by the pilot project “Biodiversity Virtual e-Laboratory” (BioVeL) (Hardisty et al. 2016), a spin-off from the LifeWatch Preparatory Phase project, is becoming a very attractive option for the expected users of LifeWatch RI. The vLabs offer high computational capacity, access to proven ready-built workflows, workflow components and other applications, capabilities for data management and a comprehensive collaborative platform that supports the needs of modern-day reproducible digital science. The latter aspects are particularly important for transparency and repeatability of the approaches followed, which forms the cornerstone of the entire scientific methodology. Finally, the LifeWatch RI may well function as an “incubation chamber” for those technologies and software developed in the context of multiple EU and nationally funded projects, which otherwise might be ceased by the end of each project.

The overall scientific challenge that LifeWatch needs to meet over its entire life-cycle is to model biodiversity on earth. The specific challenges are to: (a) reliably and continuously collect, store, manage, process, analyze and disseminate data on various biodiversity variables, such as diversity, biomass, productivity, coupled with the relevant socio-economic ones; (b) analyze the patterns, processes and consequences from possible changes on biodiversity and society welfare or to provide virtual labs offering comparable ways to asses environmental changes and risks (Los 2010); (c) offer prognosis, on for example biodiversity composition, distribution and functioning under certain scenarios (Basset and Los 2012). LifeWatch may eventually also offer support for delivering data products and other information related to Essential Biodiversity Variables (Kissling et al. 2015).

The participating MSs to the LifeWatch RI have already founded three major VREs: for marine, terrestrial and fresh water (under development) biodiversity data and data observatories. The ambition of the LifeWatch ERIC is to change the current way scientists work and think by facilitating the transition from working exclusively on their desktop personal computers to the daily use of the VREs with on-line collegial interaction. It is anticipated that this change in culture will be as disruptive as changes caused by jet traveling and internet communicating.

## Lif﻿eWatchGreece R﻿I

Greece participated to the LifeWatch preparatory phase (2008-2011) and published a request for proposals in 2009 regarding the creation of the national Research Networks in disciplines relevant to the ESFRI RIs. The Hellenic Centre for Marine Research (HCMR) - Institute of Marine Biology, Biotechnology and Aquaculture (IMBBC) has led a Consortium of 48 Research Centers and Academic Institutes, named as “Hellenic Network for Biodiversity Research” (HELBIONET). This Network represents more than 400 scientists working on various aspects of biodiversity in Greece and abroad. The main outcome of HELBIONET has been a feasibility study that confirmed that such a national Network on Biodiversity Research is both doable and viable. The subsequent construction and operation phase has been funded by the Greek General Secretariat of Research and Technology (GSRT) under the National Strategic Reference Framework (NSRF) (ESFRI, MIS 384676) which established the national LifeWatch RI, the LifeWatchGreece, which is as well coordinated by HCMR (IMBBC). The Foundation for Research and Technology Hellas (FORTH, Institute of Computer Science) has also participated in LifeWatchGreece RI as a partner while another 46 Research Institutes and Academic Departments participated as associated partners.

The overall objective of LifeWatchGreece is to develop and fulfill the vision for the Greek LifeWatch RI and to establish it as the biodiversity Centre of Excellence for South-eastern Europe, contributing with specific services to the European LifeWatch and other international users. The specific objectives are: (a) To ally all the Greek scientific human potential working on biodiversity data and data observatories, in the country and abroad, in order to achieve a world-class excellence national LifeWatch Centre. This LifeWatch Centre has to create exemplar management structures, to be legally registered, and to build the adequate interface for collaborative schemes for its continuation after the end of the national funding; (b) To pave the way for the development of complex virtual domains through a number of background e-Services that facilitate both the data contributors and the users, and which virtually ally the dynamic teams that will be continuously collecting data at the entire territory of the country; (c) To develop a number of virtual labs (vLabs) as contribution to LifeWatch Europe where large scale science can be carried out at all possible levels of the biological organization from molecules to ecosystems; (d) To build capacity at the national level through a network of activities, including human potential mobilization, supporting and promoting the use of the RI, and enhancing organizational development, and (e) To disseminate information, scientific knowledge and experience gained to the public and to liaise the Network’s ideas and practices to the activities of targeted groups and of the society at large.

The LifeWatchGreece RI is based on three simple principles: (a) Clarity: The overall and the specific objectives are clear and self-explanatory; (b) Scientific soundness and entirety: a suite of activities that facilitate the achievement of the overall and specific objectives by the Network has been implemented. These activities are interlinked and result in a number of deliverables through which both the GSRT and the Management Authority for the Operational Programme “Competitiveness and Entrepreneurship” can directly assess the performance of the Network. These deliverables are specifically designed to allow the assessment of the scientific soundness and integrity of the activities of the Network in a measurable way, and to compare them with those produced by similar Networks working on other RIs registered on the ESFRI’s roadmap; (c) Cohesion: the LifeWatchGreece RI is designed and developed in order to achieve maximum cohesion with the Thematic Area of Environment and the EU LifeWatch RI.

**Figure 1.**
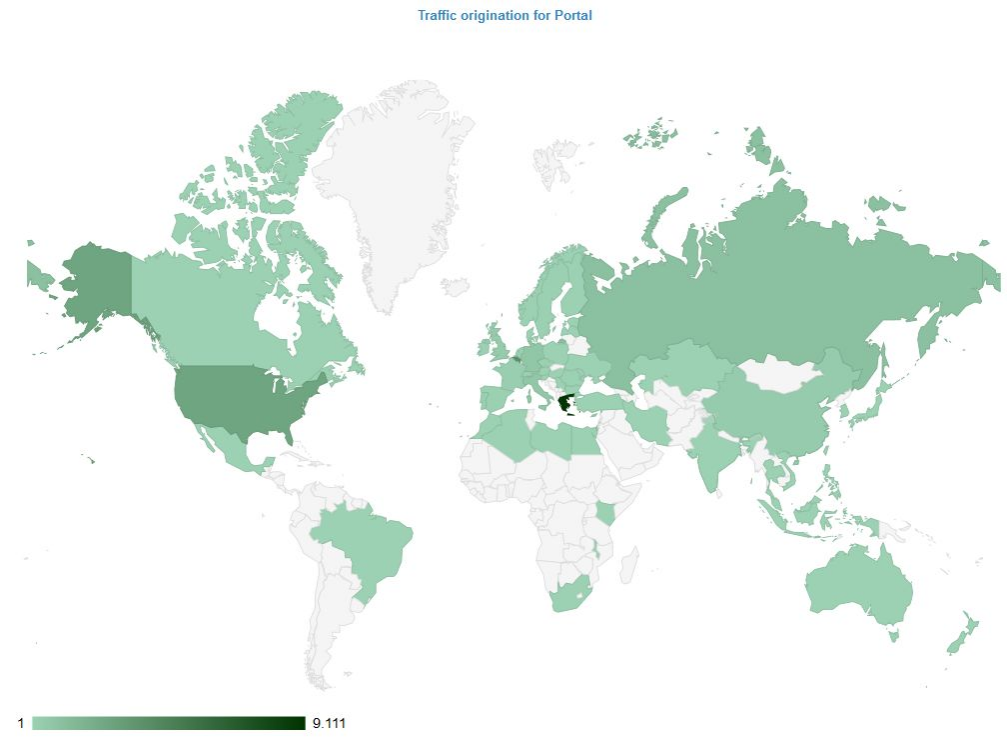
Geographic distribution of page requests received for the LifeWatchGreece web services during the academic year 2015-2016.

Only during the academic year 2015-2016, LifeWatchGreece web services received more than 9,000 page requests by users from all over the world (Fig. 1). The RI has also been used by a number of EU funded projects such as MAPMED, CIGESMED, EMBOS, ACTIONMED and EUBON. After the establishment of the LifeWatch ERIC, users can also access the services developed by LifeWatchGreece through the common LifeWatch portal.

## The Special Collection of LifeWatchGreece papers

The availability and applicability of the developed e-Services and vLabs of the LifeWatchGreece RI need to be effectively communicated to the scientific and academic society. Therefore, a series of peer reviewed scientific publications have been combined together and are presented in this LifeWatchGreece Special Collection Part 1, which is organized in four sections. The selection of Biodiversity Data Journal (BDJ) as a community peer-reviewed, open-access, comprehensive online platform for publishing part of the up-to-date outcomes of LifeWatchGreece RI, enables the publication of a wide variety of papers (e.g., software descriptions, data papers, taxonomic checklists and research articles) along with the accompanying datasets and supporting material.

The first section of papers in this special collection includes publications describing the development of **electronic infrastructure and the software applications**, such as (a) the LifeWatchGreece Portal where all the e-Services and the vLabs of the LifeWatchGreece RI are hosted; (b) the Data-Services based on Semantic Web Technologies, which are providing detailed and specialized search paths; (c) the R vLab which can be used for a series of statistical analyses in ecology (i.e., univariate and multivariate analyses, biodiversity indices), based on an integrated and optimized (in respect to computational speed-up and data manipulation) online R environment; and (d) the Micro-CT vLab, which allows the online exploration, dissemination and interactive manipulation of micro-tomography datasets.

The second section of papers includes a series of **taxonomic checklists** (preliminary, updated and/or annotated) compiled under the Greek Taxon Information System (GTIS), an initiative of LifeWatchGreece RI aiming to create a taxonomic backbone of all species reported from the Greek territory. The general guidelines, workflow and vision of the GTIS initiative are presented in an introductory paper in this special collection. All groups of biota will be progressively listed and published under GTIS while the final data, structure and tools of the GTIS database, which is under development, will be disseminated in the LifeWatchGreece portal.

The third section of papers includes a series of **data papers** that are presenting historical datasets from emblematic research expeditions, zoological collections, and original datasets of environmental and biodiversity data, covering a variety of habitats (e.g., lagoons, rocky reefs, ecosystem-engineering species and terrestrial ecosystems) and taxa (macroalgae, molluscs, polychaetes, fish etc.). This section is introduced by a special paper on the data policies adopted and implemented by the LifeWatchGreece RI.

The LifeWatchGreece RI has the capacity to provide the means, expertise and technical support for the development and maintenance of electronic platforms, as well as the analyses and interpretation of biodiversity data, using the developed vLabs, web and mobile applications. Therefore, the forth section of papers of this special collection includes a selection of original **research articles** presenting the outcomes, methodologies and citizen science initiatives developed by collaborating research projects (e.g., CIGESMED for Divers, COMBER) which shared human, hardware and software resources with LifeWatchGreece RI.

The multidisciplinary approach of LifeWatchGreece is related with the wide range of the different functionalities this RI offers. It aims to support the potential needs regarding the mobilization, analysis and sharing of biodiversity datasets, not only for the scientific community, but also for the broader domain of biodiversity management. Several initiatives under the LifeWatchGreece RI are still ongoing, continuously expanding the network of collaborations with the Greek, European and World scientific community. The construction and operation phase of this RI thus far is the initial step for the massive digitization and dissemination of several types of datasets for some of which their existence was previously unknown. The vLabs comprise mechanisms for the management of the datasets in a user friendly way that constantly attracts more users to sign in and deposit their own data. The transformation of classic research into a cyber discipline offers an easy and free (or low cost) dissemination tool for the benefit of the scientific community. The already designed after-plan for the next phase of LifeWatchGreece RI is supported by the national authorities thus ensuring the long term maintenance and updating of the Infrastructure. The continuation of its activities is also supported by the Institute of Marine Biology, Biotechnology and Aquaculture (IMBBC) of HCMR. The involved specialized staff is able to support the needs of the scientific community by providing continuous updates and developments and by enhancing the sustainability and durability of the LifeWatchGreece RI as a contribution to the LifeWatch ERIC. The design of innovative online tools and the already established IT infrastructure constitute a long term investment for the LifeWatchGreece RI. The outcomes of LifeWatchGreece have been also communicated in several national and international fora, thus attracting more users and potential investors who will offer support and ensure the LifeWatchGreece capacity in the future.
